# Involvement of DHH and GLI1 in adrenocortical autograft regeneration in rats

**DOI:** 10.1038/s41598-018-32870-9

**Published:** 2018-09-28

**Authors:** Nae Takizawa, Susumu Tanaka, Souichi Oe, Taro Koike, Takashi Yoshida, Yukie Hirahara, Tadashi Matsuda, Hisao Yamada

**Affiliations:** 1 Department of Anatomy and Cell Science, Kansai Medical University, Hirakata, Osaka 573-1010 Japan; 2 Department of Urology and Andrology, Kansai Medical University, Hirakata, Osaka 573-1010 Japan

## Abstract

Bilateral adrenalectomy forces the patient to undergo glucocorticoid replacement therapy and bear a lifetime risk of adrenal crisis. Adrenal autotransplantation is considered useful to avoid adrenal crisis and glucocorticoid replacement therapy. However, the basic process of regeneration in adrenal autografts is poorly understood. Here, we investigated the essential regeneration factors in rat adrenocortical autografts, with a focus on the factors involved in adrenal development and steroidogenesis, such as Hh signalling. A remarkable renewal in cell proliferation and increase in *Cyp11b1*, which encodes 11-beta-hydroxylase, occurred in adrenocortical autografts from 2–3 weeks after autotransplantation. Serum corticosterone and adrenocorticotropic hormone levels were almost recovered to sham level at 4 weeks after autotransplantation. The adrenocortical autografts showed increased *Dhh* expression at 3 weeks after autotransplantation, but not *Shh*, which is the only Hh family member to have been reported to be expressed in the adrenal gland. Increased *Gli1* expression was also found in the regenerated capsule at 3 weeks after autotransplantation. *Dhh* and *Gli1* might function in concert to regenerate adrenocortical autografts. This is the first report to clearly show *Dhh* expression and its elevation in the adrenal gland.

## Introduction

The adrenal gland comprises the adrenal cortex and the medulla. In humans, the adrenal cortex is composed of three anatomical layers namely the zona glomerulosa (ZG), zona fasciculata (ZF), and zona reticularis. In the rat adrenal cortex, aldosterone synthase (*Cyp11b2*) in ZG and 11-beta-hydroxylase (*Cyp11b1*) in ZF catalyse the production of aldosterone and corticosterone, respectively. These CYP genes are regulated by NR5A1 (also known as SF1 or Ad4BP)^1^. There is also an area with no expression of *Cyp11b1* and *Cyp11b2* termed the undifferentiated zone (ZU) located between ZG and ZF in rats^2–4^.

Familial phaeochromocytoma patients, such as those with multiple endocrine neoplasia type 2, may require bilateral adrenalectomy, after which the patients need to take glucocorticoid replacement therapy throughout their life^5^. Moreover, the risk of adrenal crisis, a life-threatening event, remains as long as the patients live^6^. Therefore, partial adrenalectomy may be performed as an adrenal sparing surgery to avoid adrenal crisis^5^. However, there is the risk that phaeochromocytoma may recur from the ipsilateral residual adrenal gland even after partial adrenalectomy^7^. In this case, autotransplantation of the adrenal cortex may reduce the risk of phaeochromocytoma recurrence^8^ because the adrenal medulla, which is regarded as the origin of phaeochromocytoma, does not regenerate, whereas adrenocortical cells can be regenerated in adrenal autografts in animal models^9^. Adrenal transplantation was studied in animals even in the early 1900s^10^; to our knowledge, the oldest reported adrenal transplantation was conducted in a patient with Addison’s disease in 1922^11^. Establishment of adrenocortical autotransplantation has the advantage of reducing recurrence risk and eliminating the need for glucocorticoid replacement therapy in familial phaeochromocytoma patients^8^. However, the success rate for adrenal autotransplantation in humans is low, ranging from 20 to 35%^12,13^ in contrast to animal models^14,15^. Consequently, to clarify the regeneration step of adrenocortical autografts after transplantation, and the factors affecting their remodelling and regeneration, analysis of adrenocortical autografts in animal models is required.

Although adrenocortical autotransplantations have been successful in rodents^14,15^, the underlying mechanism has not been clarified yet. Several patterns of adrenocortical cell zonation, renewal, and remodelling under various conditions have been reported^2,16,17^. Apparently, pools of stem/progenitor cells exist in the adrenal capsule, the subcapsular, and other regions of the adrenal gland^2,18^. It is well-known that SHH plays a key role in adrenal development and is localised in the subcapsular region of mice and in the ZU of rats^3,18,19^. *Shh*(+) cells have also been identified as stem/progenitor cells^2,18–20^. To date, only *Shh* has been reported to be expressed in the adrenal gland among the Hh family members^2^. SHH regulates *Gli1*, the effector of Hh signalling and a determinant of cell fate during embryonic development^21^. *Gli1*(+) cells have also been identified as stem/progenitor cells and are localised in the adrenal capsule^22^. In addition, SHH(+) and GLI1(+) progenitor cells differentiate into steroidogenic cells. It is conceivable that the Hh signalling pathway is involved in the regeneration process of adrenocortical autografts as in the development of the adrenal cortex. In this study, we examined whether Hh signalling functions as a remodelling and/or regeneration factor for adrenocortical autografts. Unexpectedly, we found that *Shh* was suppressed and *Dhh* was upregulated in the regeneration step of rat adrenocortical autografts. Our study clearly demonstrates *Dhh* expression in the adrenal gland for the first time.

## Results

### Enlargement of adrenocortical autografts

Macroscopically (Supplementary Fig. 1), we identified the adrenocortical autograft as a yellowish mass throughout the post-operative period. Adrenocortical autografts showed thin shapes at post-operative day (POD) 7 (Supplementary Fig. 1). At two weeks after surgery, almost all adrenocortical autografts were flat-shaped and fragile to handle in all rats (Supplementary Fig. 1). After three weeks (Supplementary Fig. 1), the autografts were stable and increased in size compared to POD14. There was no difference between POD21 and POD28 with respect to gross appearance.

**Figure 1:**
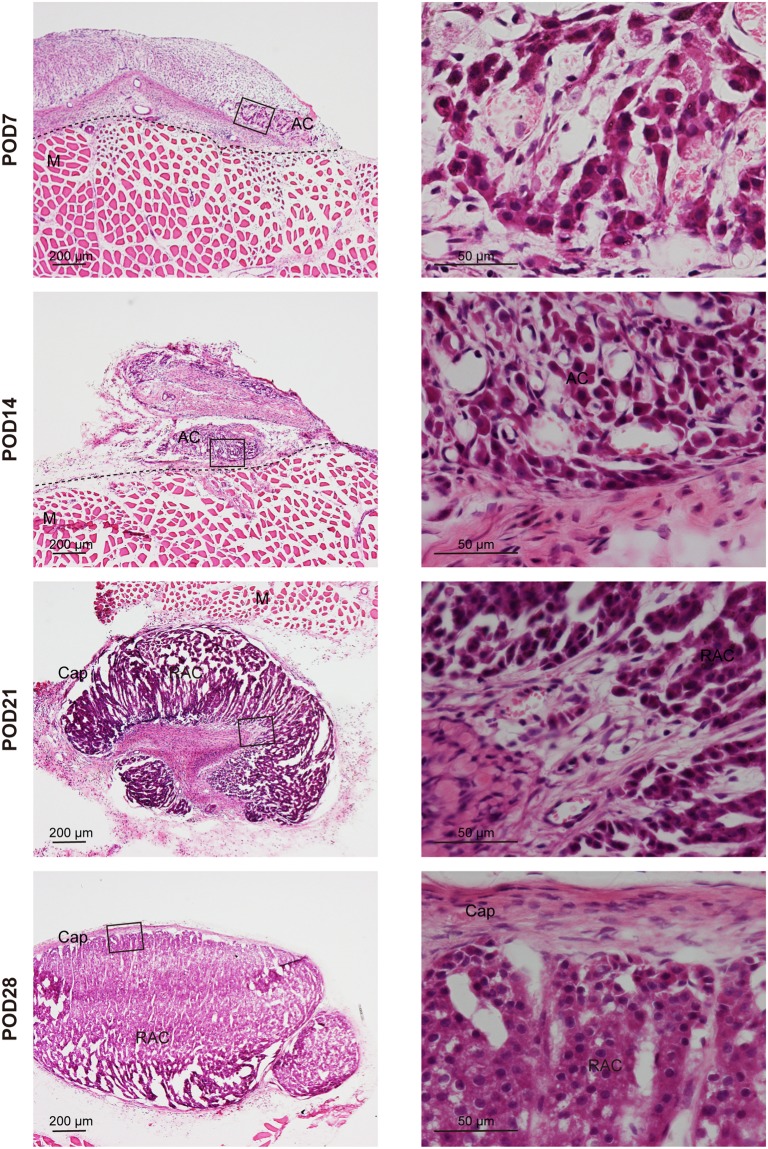
Morphological changes in adrenocortical autografts. Microscopic findings of adrenocortical autografts. Left panels show the low-power fields of HE-stained sections. Right panels are the expanded views of the square fields in the left panel. Sections on post-operative day (POD) 7 and POD14 showed only a few remnant adrenocortical cell clusters over adrenocortical autografts. An obvious renewal capsule and renewal adrenocortical cell cluster were detected at POD21 and POD28. Cap: capsule; AC: adrenocortical cells; RAC: renewal adrenocortical cells; M: muscle. The black broken line shows the boundary between the adrenocortical autograft and muscle tissue.

Haematoxylin and eosin (HE) staining showed (i) partial loss of adrenocortical cells, (ii) a few remnant adrenocortical cell clusters around the capillaries, and (iii) stromal-like cell proliferation from POD7 to POD14 (Fig. 1). At POD21, we easily identified the arrangement of the renewing adrenocortical cells in the cord-like structures similar to the normal ZF (Fig. 1). These cord-like structures were completely encapsulated in the renewal capsule and in the columns directed towards the veins (Supplementary Fig. 2). Indeed, a prominent increase in adrenocortical cells was observed in the region encapsulated within the capsule after POD16 (Fig. 1 and Supplementary Fig. 2). Four weeks after surgery, HE staining indicated fully formed adrenocortical cells (Fig. 1).

### Autotransplanted rats recover from Adrenocortical insufficiency at POD21

**Figure 2:**
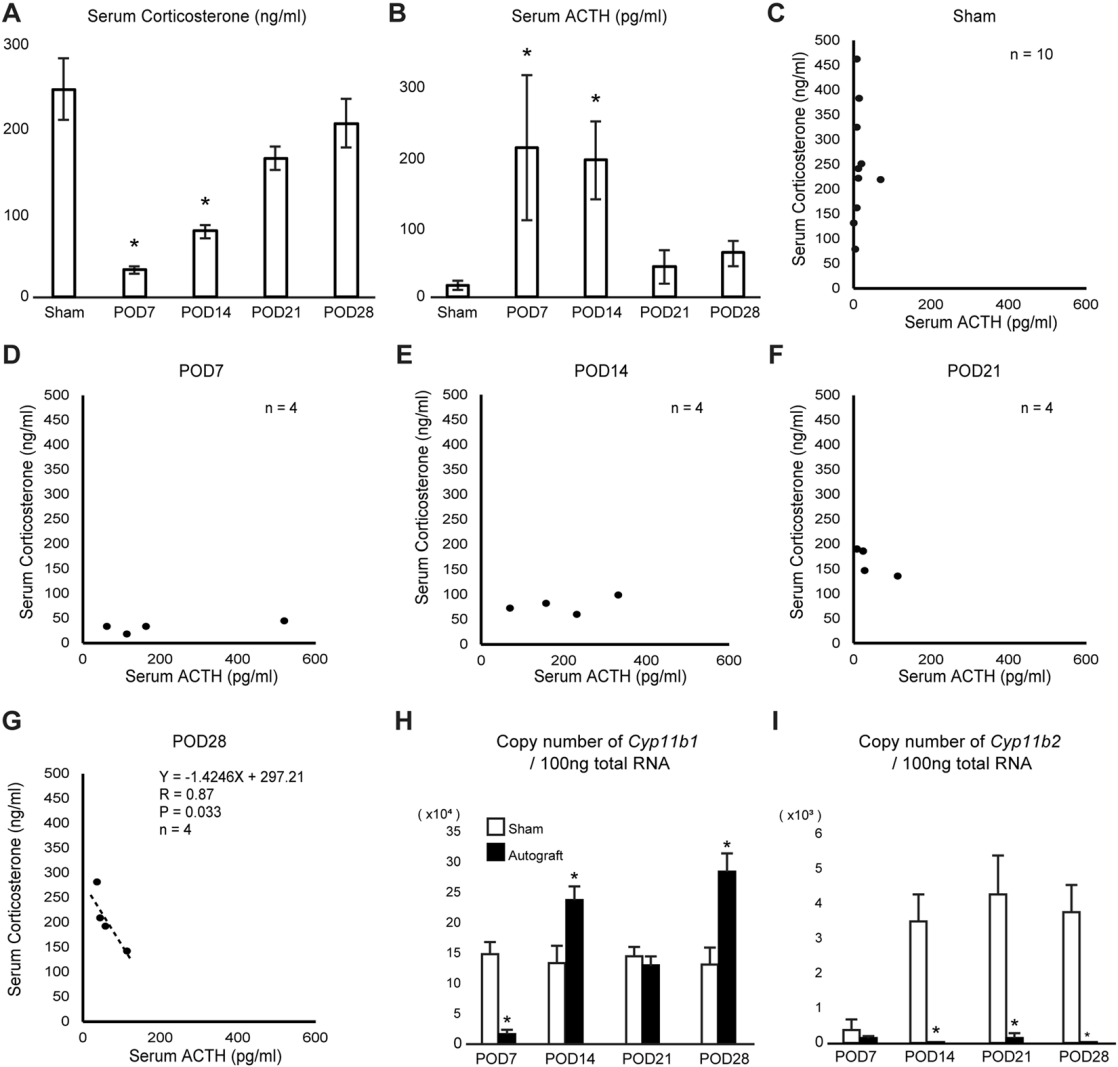
Change of endocrine function in adrenocortical autografts. (**A**,**B**) Serum corticosterone and ACTH levels in rats (n = 10 in sham group, n = 4 per autografted group). *p < 0.0125 *vs*. sham group. Each value represents the mean ± standard error. (**C**–**G**) The correlation between corticosterone and ACTH at each time point. Serum ACTH levels show significantly negative correlation with corticosterone levels at POD 28 (P = 0.033, R = 0.87). (**H**,**I**) Detection of *Cyp11b1* and *Cyp11b2* in adrenocortical autografts using nCounter technology. The mean expression at each time point in adrenocortical autografts was compared with that in the sham-operated adrenal gland. *p < 0.05 vs. sham operation at each time point. The white bar indicates the copy number of each mRNA in the sham group and the black bar indicates the same in autografts. Values are presented as the mean + standard deviation.

Serum corticosterone (ng/ml) showed a significant reduction at POD7 (n = 4, mean ± standard error; 32.6 ± 5.3, p = 0.002, r = 0.74) and POD14 (n = 4, 78.4 ± 8.0, p = 0.008, r = 0.67) compared to the sham group (n = 10, 246.9 ± 33.5) (Fig. 2A). There was a gradual increase in the level of serum corticosterone. There was no significant difference between the sham group and POD21 (n = 4, 164.9 ± 13.7) or the sham group and POD28 (n = 4, 206.4 ± 28.8). On the other hand, serum adrenocorticotropic hormone (ACTH) (pg/ml) showed a significant increase at POD7 (n = 4, mean ± standard error; 215.3 ± 103.7, p = 0.004, r = 0.72) and POD14 (n = 4, 197.1 ± 55.7, p = 0.004, r = 0.72) compared to the sham group (n = 10, 17.1 ± 6.2) (Fig. 2B). There was no significant difference between the sham group and POD21 (n = 4, 44.1 ± 23.9) or the sham group and POD28 (n = 4, 63.8 ± 17.5).

To clarify the recovery of the adrenocortical insufficiency in adrenal autografts, the correlations between corticosterone and ACTH were analysed at each time point (Fig. 2C–G). A significant negative correlation was found between corticosterone and ACTH at POD28 (r = 0.86, p < 0.05) (Fig. 2G). Although normal values of serum corticosterone and improvement of the hyperactive hypothalamic-pituitary-adrenal axis were found at POD28, latent adrenocortical insufficiency was suggested at POD28 based on the negative correlation.

### Increase in *Cyp11b1* (+) cells in adrenocortical autografts

To confirm the evidence of functional recovery, we examined *Cyp11b1* expression in adrenocortical autografts using the RNAscope/Basescope technique.

RNAscope is a novel RNA *in situ* hybridisation analysis using a unique double-Z design probe^23^. The design helps to increase the sensitivity and specificity for target gene expression. RNAscope signals show as dots, with each dot corresponding to a single mRNA copy^23^.

**Figure 3:**
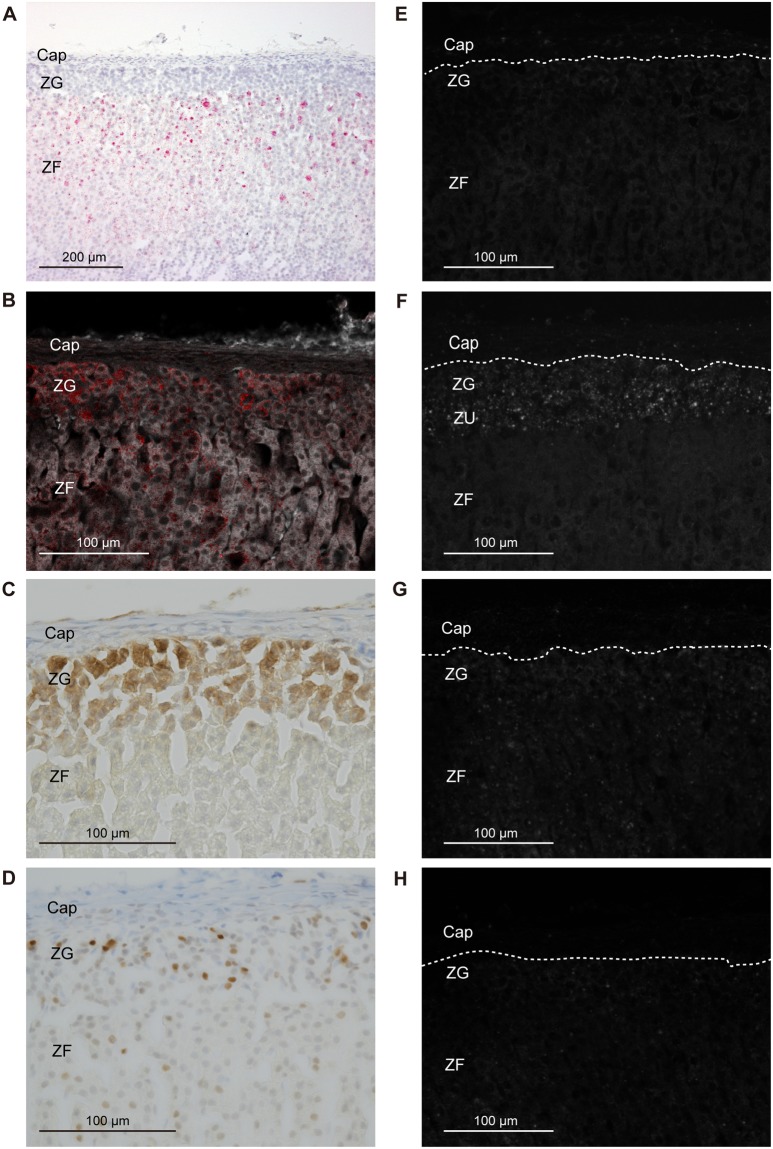
IHC and *in situ* hybridisation in adrenal gland with sham operation. (**A**) *Cyp11b1* expression with *in situ* hybridisation using Basescope. *Cyp11b1* was detected in the zona fasciculata of the sham-operated adrenal glands. (**B**) CYP11B2 expression with IHC. CYP11B2 signals are indicated as red and autofluorescence as white. (**C**) DAB2 expression with IHC. (**D**) PCNA expression with IHC. (**E**) *Gli1* with *in situ* hybridisation using RNAscope. (**F**) *Shh* with *in situ* hybridisation using RNAscope. (**G**) *Dhh* with *in situ* hybridisation using RNAscope. (**H**) *Ihh* with *in situ* hybridisation using RNAscope. In panels E–H, one dot signal represents one mRNA copy in the RNAscope results. RNAscope signals are represented as white colour and autofluorescences were subtracted from the original figures (Supplementary Fig. 3). The white broken line shows the boundary between the adrenal capsule and adrenocortical cells. Cap: capsule; ZG: zona glomerulosa; ZF: zona fasciculate; ZU: undifferentiated zone.

**Figure 4:**
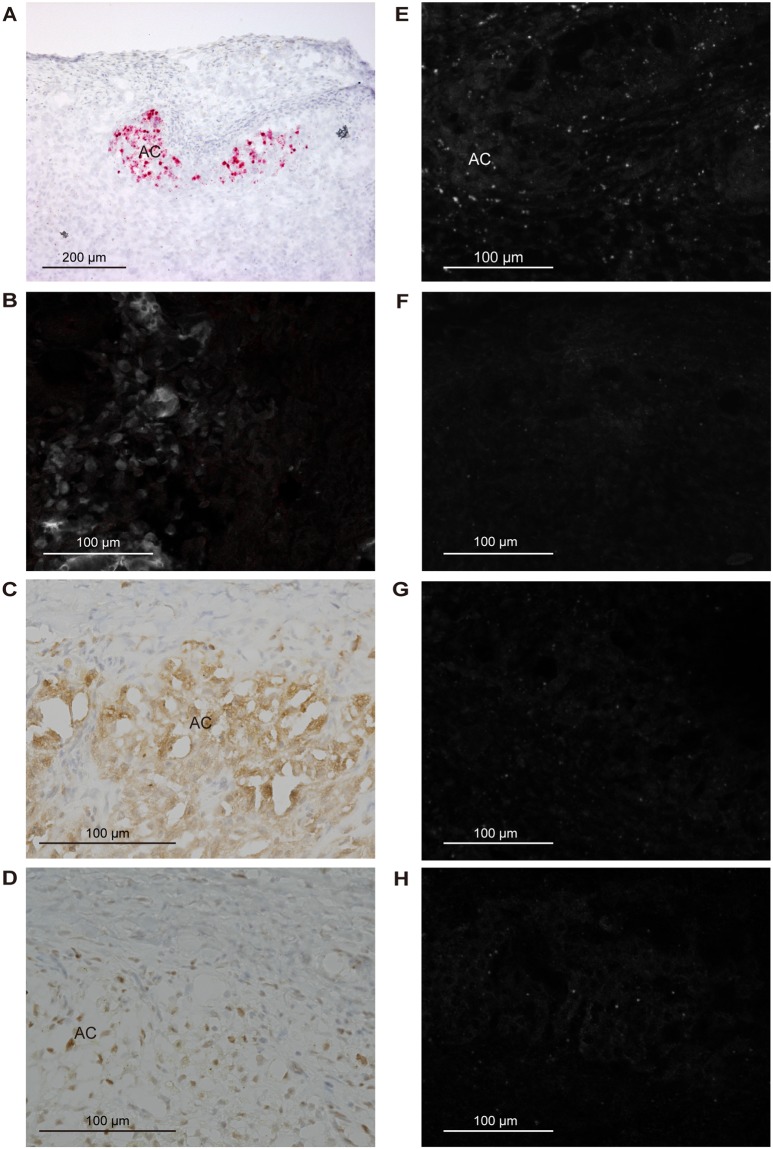
IHC and *in situ* hybridisation in the adrenocortical autograft at POD7. (**A**) *Cyp11b1* expression with *in situ* hybridisation using Basescope. *Cyp11b1* was detected in remnant adrenocortical cells. (**B**) CYP11B2 expression with IHC. CYP11B2 signals are indicated as red and autofluorescence as white. No CYP11B2 was found. (**C**) DAB2 expression with IHC. (**D**) PCNA expression with IHC. (**E**) *Gli1* with *in situ* hybridisation using RNAscope. (**F**) *Shh* with *in situ* hybridisation using RNAscope. (**G**) *Dhh* with *in situ* hybridisation using RNAscope. (**H**) *Ihh* with *in situ* hybridisation using RNAscope. In panels E–H, one dot signal represents one mRNA copy in the RNAscope results. RNAscope signals are represented as white colour dots and autofluorescences were subtracted from original figures (Supplementary Fig. 3). AC: adrenocortical cells.

**Figure 5:**
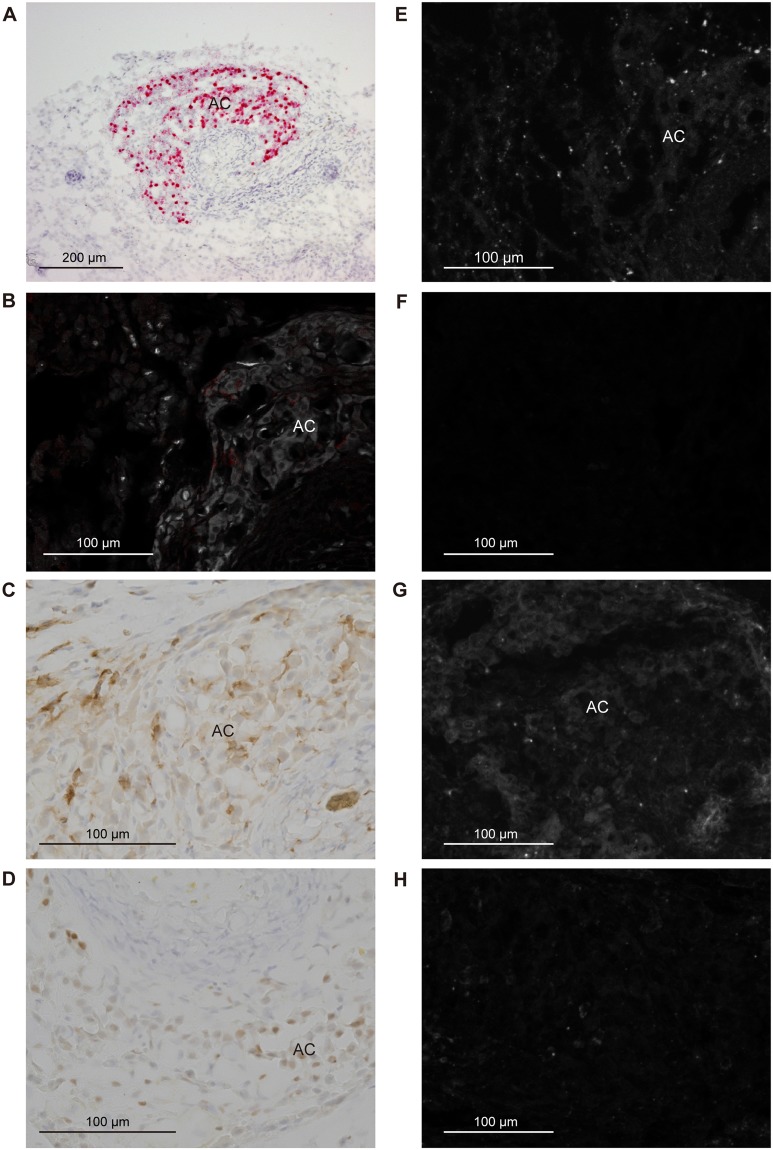
IHC and *in situ* hybridisation in the adrenocortical autograft at POD14. (**A**) *Cyp11b1* expression with *in situ* hybridisation using Basescope. *Cyp11b1* was detected in remnant adrenocortical cells. (**B**) CYP11B2 expression with IHC. CYP11B2 signals are indicated as red and autofluorescence as white. No CYP11B2 was found. (**C**) DAB2 expression with IHC. (**D**) PCNA expression with IHC. (**E**) *Gli1* with *in situ* hybridisation using RNAscope. (**F**) *Shh* with *in situ* hybridisation using RNAscope. (**G**) *Dhh* with *in situ* hybridisation using RNAscope. (**H**) *Ihh* with *in situ* hybridisation using RNAscope. In panels E–H, one dot signal represents one mRNA copy in the RNAscope relults. RNAscope signals are represented as white colour dots and autofluorescences were subtracted from original figures (Supplementary Fig. 3). AC: adrenocortical cells.

**Figure 6:**
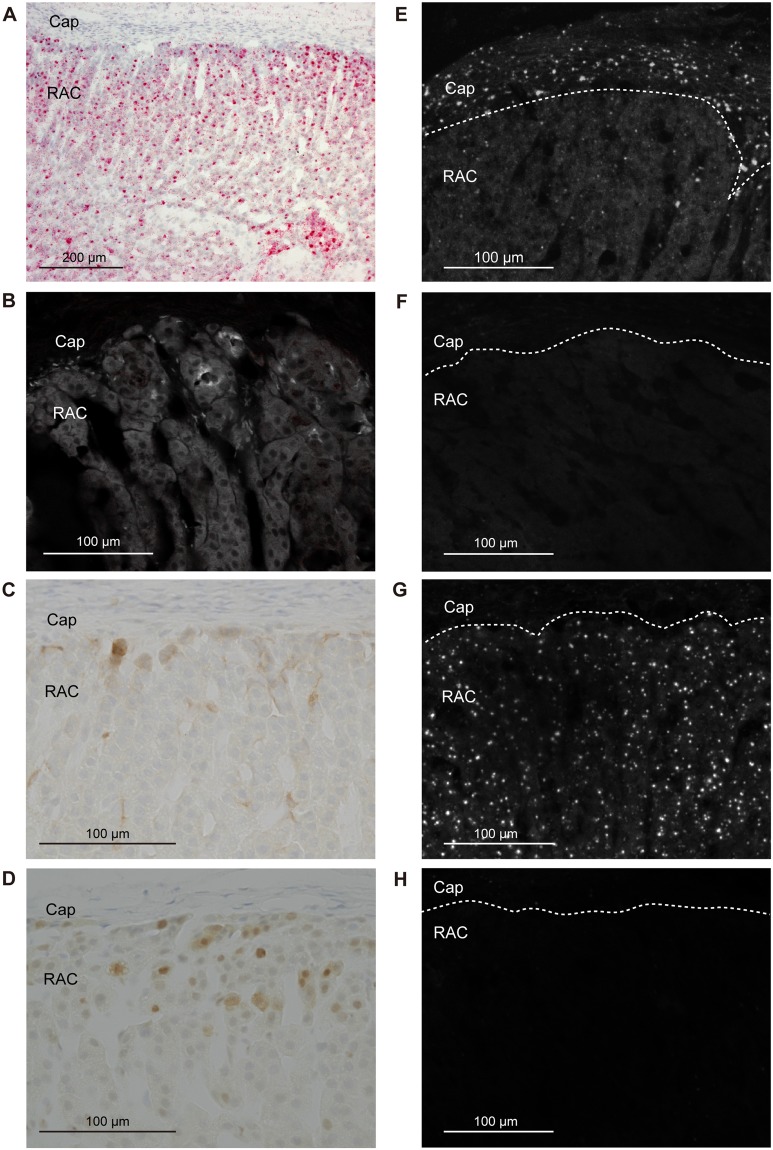
IHC and *in situ* hybridisation in the adrenocortical autograft at POD21. (**A**) *Cyp11b1* expression with *in situ* hybridisation using Basescope. *Cyp11b1* was detected in renewal adrenocortical cells. (**B**) CYP11B2 expression with IHC. CYP11B2 signals are indicated as red and autofluorescence as white. No CYP11B2 was found. (**C**) DAB2 expression with IHC. DAB2 was detected in subcapsular region. (**D**) PCNA expression with IHC. (**E**) *Gli1* with *in situ* hybridisation using RNAscope. (**F**) *Shh* with *in situ* hybridisation using RNAscope. (**G**) *Dhh* with *in situ* hybridisation using RNAscope. (**H**) *Ihh* with *in situ* hybridisation using RNAscope. In panels E–H, one dot signal represents one mRNA copy in the RNAscope results. RNAscope signals are represented as white colour dots and autofluorescences were subtracted from original figures (Supplementary Fig. 3). Cap: capsule; RAC: renewal adrenocortical cells.

**Figure 7:**
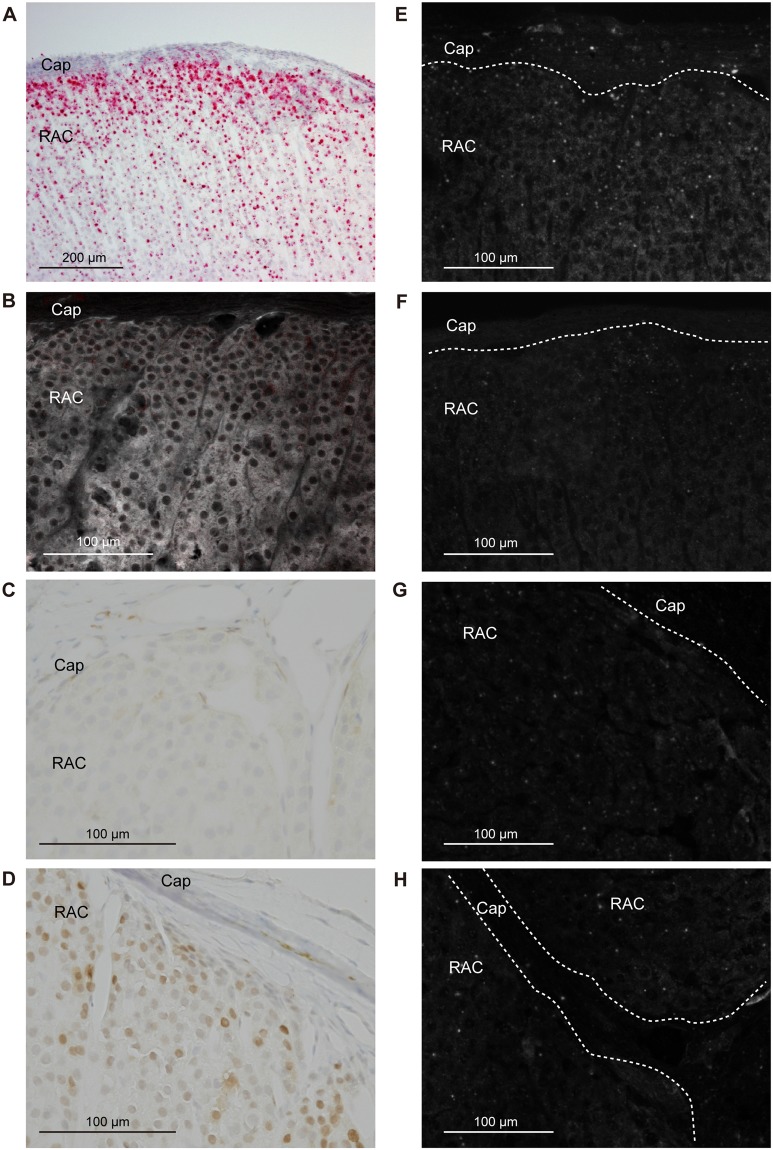
IHC and *in situ* hybridisation in the adrenocortical autograft at POD28. (**A**) *Cyp11b1* expression with *in situ* hybridisation using Basescope. *Cyp11b1* was detected in renewal adrenocortical cells. (**B**) CYP11B2 expression with IHC. CYP11B2 signals are indicated as red and autofluorescence as white. No CYP11B2 was found. (**C**) DAB2 expression with IHC. DAB2 was detected in subcapsular region. (**D**) PCNA expression with IHC. (**E**) *Gli1* with *in situ* hybridisation using RNAscope. (**F**) *Shh* with *in situ* hybridisation using RNAscope. (**G**) *Dhh* with *in situ* hybridisation using RNAscope. (**H**) *Ihh* with *in situ* hybridisation using RNAscope. In panels E–H, one dot signal represents one mRNA copy in the RNAscope results. RNAscope signals are represented as white colour dots and autofluorescences were subtracted from original figures (Supplementary Fig. 3). Cap: capsule; RAC: renewal adrenocortical cells.

*Cyp11b1* staining in the adrenal gland of a sham-operated rat using the Basescope showed a relatively strong expression in the ZF and a gradually decreasing expression towards the inner medulla (Fig. 3A). In adrenocortical autografts at POD7 and POD14, remnant adrenocortical cells around the capillary circumference showed strong *Cyp11b1* expression (Figs 4A and 5A). There was no signal in the autografts except for remnant adrenocortical cells at POD7 and POD14 (Figs 4A and 5A). Diffuse *Cyp11b1* expression over the adrenocortical autograft was recognised at POD21 (Fig. 6A), and the expression level per cell was lower than that in the adrenocortical autograft at POD28 (Fig. 7A). At POD28, *Cyp11b1* was expressed in a polarised manner, which is similar to its expression pattern in the adrenal gland of sham-operated rats, and a strong *Cyp11b1* expression was detected in the regenerated ZF-like region just beneath the renewing capsular region (Fig. 7A).

### Recovery of *Cyp11b1* and absence of *Cyp11b2/*CYP11B2 in adrenocortical autografts

To confirm the expression of *Cyp11b1* and *Cyp11b2* in whole adrenocortical autografts, we conducted digital mRNA counts using nCounter technology (Fig. 2H,I). The *Rattus Cyp11b1* (Acc#: NM_012537.3) sequence contains several deletions compared with the *Rattus Cyp11b2* sequence (NM_012538.2); therefore, we created a unique probe against *Rattus Cyp11b2*, but we could not create a unique probe for *Cyp11b1*, because the homology among *Rattus Cyp11b1, Cyp11b2* (94% identity against Cyp11b1)*, Cyp11b3* (NM_181824.1, 94% identity), and *Cypxib1* (XM_017595365.1) is very high.

No expression of *Cyp11b2* was found with the specific *Cyp11b2* probe in adrenocortical autografts (mean ± standard deviation, 76.4 ± 76.1 at POD7, 19.7 ± 5.6 at POD14, 117.4 ± 152.9 at POD21, 8.8 ± 5.8 at POD28) (Fig. 2I), as less than 120 counts with the *Cyp11b2* probe are considered negative depending on the background counts. *Cyp11b2* was expressed in the sham-operated adrenal gland (357.2 ± 310.4 at POD7, 3506.6 ± 754.0 at POD14, 4268.8 ± 1133.9 at POD21, 3758.7 ± 781.1 at POD28). Saline supplementation might have suppressed the *Cyp11b2* expression at POD7 (see Materials and Methods). In contrast, the *Cyp11b1* probe in nCounter detected a significant reduction in cytochrome P450 11B mRNAs in the autografts (17313.8 ± 5046.9) compared with that in sham animals (146606.1 ± 19691.6) at POD7 (p = 0.021, *r* = 0.82) and demonstrated upregulation of cytochrome P450 11B mRNAs in the autografts (236321.2 ± 20738.8) compared with that in sham animals (131732.2 ± 29101.7) at POD14 (p = 0.021, *r* = 0.82) (Fig. 2H). We had estimated that *Cyp11b1* expression was decreased at POD7 and POD14, which reflected a loss of adrenocortical cells as determined using the Basescope. However, *Cyp11b1* expression did not result in a gradual increase in serum corticosterone levels and had a transient peak at POD14, suggesting that the nCounter *Cyp11b1* probe might have detected other cytochrome P450 11B mRNAs. At POD21, there was no significant difference in the detection of the nCounter *Cyp11b1* probe between the autografts (128927.5 ± 14906.7) and sham animals (147918.0 ± 12235.6). The nCounter *Cyp11b1* probe detected a significant increase in cytochrome P450 11B mRNAs in the autografts (283069.0 ± 29285.4) compared with that in sham animals (132757.8 ± 24891. 9) at POD28 (p = 0.021, *r* = 0.82).

To examine the presence of ZG cells in the adrenocortical autograft, we immunohistochemically stained for rattus ZG specific markers, CYP11B2 and DAB2^24,25^. CYP11B2 staining in the adrenal gland of a sham-operated rat showed a strong expression in the ZG (Fig. 3B). The absence of CYP11B2 was observed in the adrenocortical autograft as well as the results of nCounter (Figs 4B–7B). However, DAB2 signals were detected not only in the ZG of sham rat, but also in remnant adrenocortical cells at POD7 and POD14 and in the subcapsular region at POD21 and POD28 (Figs 3C–7C).

### Adrenocortical autograft cell proliferation occurs in subcapsular region

To identify the cell proliferation in adrenal autografts, proliferating cell nuclear antigen (PCNA) staining was performed. Cell proliferation has been reported to be dominant in ZG in the adrenal gland,^26^ and PCNA signals in adrenal cortex with sham operation were also found in ZG (Fig. 3D). At POD7 and POD14, PCNA was broadly and sparsely observed in autografts (Figs 4D and 5D). PCNA was detected in subcapsular region again at POD21 and POD28 (Figs 6D and 7D).

### Upregulation of *Dhh* in adrenocortical autografts

In the adrenal cortex of sham-operated rats, RNAscope findings for *Gli1* and *Shh*, which were detected in the adrenal capsule and in the ZU, respectively (Fig. 3E,F), were consistent with a previous report^27^. *Gli1* was expressed in stromal cells adjacent to the remnant adrenocortical cells at POD7 and POD14 (Figs 4E and 5E). *Gli1* expression sites were changed to renewal capsular cells and adrenocortical cells close to the capsule at POD21 (Fig. 6E). *Gli1* expression at POD28 was decreased in the renewal capsular region compared to that at POD21 (Figs 6E and 7E), and persisted in the adrenocortical cells in the subcapsular region at POD28 (Fig. 7E). We did not detect any *Shh* expression through POD7–21 (Figs 4F–6F). We detected a few *Shh* signals in the regenerated adrenocortical cells at POD28 (Fig. 7F). In contrast, *Dhh* was remarkably and diffusely expressed in the renewal adrenocortical cells at POD21 compared to the sham-operated adrenal gland (Figs 3G and 6G). A few *Dhh* expression signals were detected at POD7 and POD14 (Figs 4G and 5G). *Dhh* showed scattered expression in both the adrenal cortex with sham operation and adrenocortical autografts at POD28 (Fig. 7G). In contrast to *Dhh* expression, few *Ihh* expression was detected in the adrenal gland upon sham operation and in adrenocortical autografts. There was also no change throughout the regeneration period (Figs 3H–7H).

The dim cytoplasmic fluorescence signals were regarded as autofluorescence of adrenocortical cells (Supplementary Fig. 3). We used unmixing analysis for extracting autofluorescence (Supplementary Fig. 4 and method section).

### A few *Wt1*(+) *Gli1*(+) cells in adrenocortical autografts

**Figure 8:**
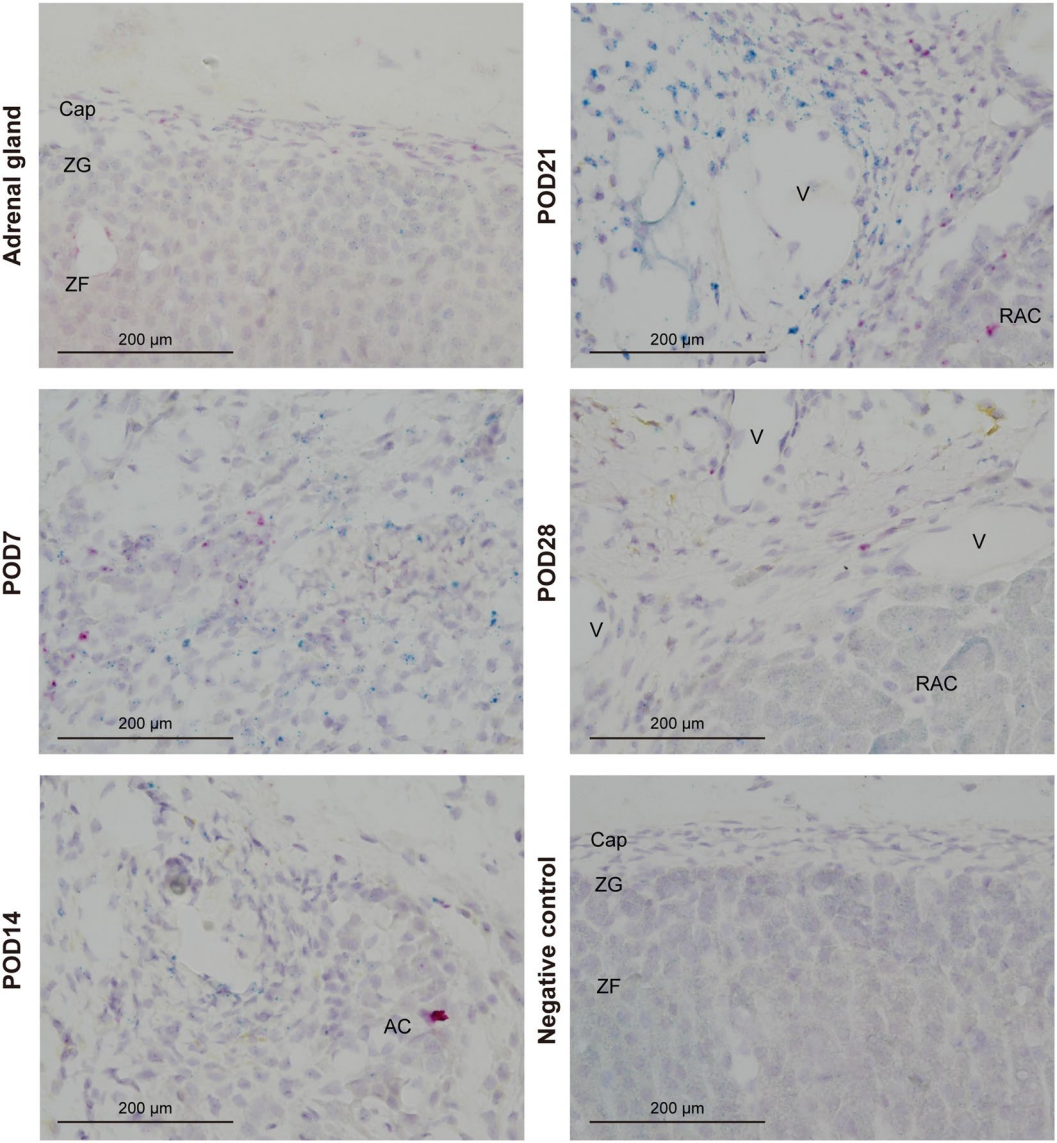
*Wt1* and *Gli1* expression in sham-operated adrenal glands and adrenocortical autografts. *Wt1* (FastGreen) and *Gli1* (FastRed) were expressed in the adrenal capsule. Sections on post-operative day (POD)7 to POD21 showed *Wt1* expression around the capillaries, and few *Wt1* (+) and *Gli1* (+) cells were found. Cap: capsule; ZG: zona glomerulosa; ZF: zona fasciculata; RAC: renewal adrenocortical cells; V: vein.

WT1 has been reported as a direct regulator of *Gli1* without Hh input in the adrenal gland^28^. To clarify the involvement of WT1 in regulating *Gli1* in the adrenocortical autograft, double staining with *Wt1* (FastGreen) and *Gli1* (FastRed) was conducted using RNAscope (Fig. 8). In the adrenal glands of sham-operated rats, *Wt1* expression was found to be weak in the capsule and cortex, and *Gli1* was located in the capsule (Fig. 8). Some blurred blue signals in adrenal cells were regarded as background based on negative control study (Fig. 8). From POD7 to POD21, we found *Wt1* expression around the capillaries, *Gli1* expression adjacent to *Wt1*, and a few *Wt1*(+) *Gli1*(+) cells (Fig. 8). *Wt1* was suppressed at POD14 and its expression was increased again around the veins at POD21 (Fig. 8). At POD28, *Wt1* was re-suppressed around the veins and slightly expressed in renewal adrenocortical cells (Fig. 8).

## Discussion

HE staining of adrenocortical autografts showed loss of most adrenocortical cells by 1 week after operation. The dynamic changes, dependent on the regeneration step, occurred between 2 and 3 weeks after surgery as reported previously^9,15,29^. After four weeks, the adrenocortical autografts showed morphological and functional recovery as shown by the restoration of serum corticosterone levels with no statistical difference compared to sham animals. We also found strong expression of *Dhh* and *Gli1* during the remodelling and regeneration process in adrenocortical autografts.

Differentiated ZG cells are known to undergo lineage conversion into ZF cells^15,16^. ZG-specific gene deletion of *Nr5a1* prevents lineage conversion into ZF cells, but the production of functional ZF cells is maintained^16^. These results suggest that ZF cells are produced from ZG cells as well as from other cell populations. There was no obvious ZG in the autograft at POD21 and POD28 based on our *Cyp11b1* and *Cyp11b2*/CYP11B2 expression analysis with immunohistochemistry (IHC), *in situ* hybridisation, and the nCounter method. The *Cyp11b1* expression pattern in the Basescope analysis and the lack of *Cyp11b2*/CYP11B2 expression in IHC and the nCounter analysis indicated that almost all the regenerated adrenocortical cells were ZF cells. Cell proliferation has been shown dominantly in both mouse ZG^26^ and ZF in rat enucleation models of the adrenal gland^30^. In the present study, PCNA (+) cells were increased at POD7 and POD14, and strong PCNA (+) cells were located in the subcapsular region at POD21 and POD28. These findings were consistent with a previous report^31^. These regenerated adrenocortical cells in the subcapsular region might be directly differentiated into *Cyp11b1*-expressing cells in the adrenocortical autografts without the lineage conversion from ZG cells.

Although no CYP11B2 was found at POD7 and POD14 indicating the absence of ZG cells, strong DAB2 signals were found in those periods. Absence of ZG in adrenocortical autograft was also previously reported^32^. DAB2 has multifaceted roles and is expressed in epithelial cells of several tissues^33^. DAB2 is also induced by physiological stimulations^34^. In addition, it is known that CYP11B2 expression is restricted to a limited number of ZG cells expressing DAB2 in humans^35^. Therefore, there might be a dissociation between CYP11B2 and DAB2 in adrenal cortex remodelling. We observed DAB2 expression in remnant adrenocortical cells at the early stages. However, subcapsular localisation of DAB2 was found on POD21. It could be said that immature ZG cells without CYP11B2 might be regenerated from POD21 onwards.

Not surprisingly, overall *Gli1* expression was observed in the adrenocortical autograft similar to the developmental stage, suggesting the participation of Hh signalling in the regeneration of adrenocortical autografts. The expression was prominent at POD21. Although *Shh* (+) *Gli1* (+) progenitor cells have been reported to differentiate into steroidogenic cells in the canonical process^18^, we observed only slight expression of *Shh* in the adrenocortical autografts with no correlation between *Shh* and *Gli1* expression in the adrenocortical autografts. We then performed RNAscope analysis with other Hh signalling ligands, *Dhh* and *Ihh*. Expression of *Ihh* was hardly found and showed no change in all the experiments. In contrast, *Dhh* showed strong and diffused expression in the regenerating adrenocortical cells at 3 weeks after the operation. DHH but not SHH might be one of the key regulators of GLI1 in adrenocortical autografts as in gonads^21,36,37^, and the upregulation of *Dhh* implies its importance for the regeneration of adrenocortical autografts.

It is well known that WT1 directly controls *Gli1* transcription in the adrenal primordia without Hh signalling^28^ and that it is implicated in steroidogenic cell differentiation in both the adrenal cortex and the gonads^28,38^. Moreover, *WT1* is a tumour suppressor gene and is related to the distant metastasis and progression of tumour cells by its function as a master regulator of the mesenchymal-epithelial transition and epithelial-mesenchymal transition^39^. *WT1* is highly expressed in the blood vessels of most human tumours but not in the blood vessels in normal tissues^40,41^. In this study, no increase in *Wt1* expression was found in the mesenchyme of normal muscle tissues at the site of transplantation or in the remnant autografted adrenal tissues at POD7. However, WT1 could be involved in graft vascularisation, as a highly specific expression of *Wt1* was observed in the perivascular regions in autografts at POD7. Interestingly, an alternative splice variant, WT1-KTS, is known to stimulate the expression of the receptor for the vascular endothelial growth factor, *Kdr*, in murine embryonic gonads^42^, and a high *Kdr* expression was observed in a previous study on adrenocortical autografts^43^. Accordingly, regulation of vascularisation by WT1 was considered as one of the key factors for the regeneration step of adrenocortical autografts at the initial stage.

In conclusion, an increase in *Dhh* and *Gli1* expression was observed during the regeneration of adrenocortical tissues. WT1 was suggested to contribute to the regeneration of blood vessels and synergistically potentiate DHH effect. This report is the first to clearly demonstrate the expression of *Dhh* and its elevation in the adrenal gland. To clarify whether DHH directly affects adrenocortical regeneration, further autotransplantation studies using *Dhh* knockout strains with genome editing will need to be conducted.

## Materials and Methods

### Animals

In total, 37 male Wistar rats (age, 7 weeks; weight, 210–270 g) were purchased from Shimizu Laboratory Supplies (Kyoto, Japan) and housed in a sound-attenuated light-controlled room (lights on at 8:00 a.m. and off at 8:00 p.m.; 12 h light-dark cycle; constant environment at 25 ± 1 °C and 50 ± 10% relative humidity) for 2 weeks before the operation. Food and water were provided *ad libitum*. All animal experiments were approved by the Ethics Committee on Animal Experiments at Kansai Medical University (approval ID: 17-051) and were conducted in accordance with the Guide for the Care and Use of Laboratory Animals of the Institute for Laboratory Animal Research.

### Adrenocortical autotransplantation

Adrenocortical autotransplantation was performed in 9-week-old rats as described previously^44^. Briefly, bilateral adrenal glands were resected under general anaesthesia by inhalation of 2% isoflurane (Pfizer Japan Inc., Tokyo, Japan) and 3 L/minute oxygen. The adrenal gland was divided into four pieces and the medulla was discarded. The adrenocortical autografts retaining the capsule, ZG, and ZU^4^ were autotransplanted into the right biceps femoris. Sham operations without adrenalectomy were performed simultaneously. The animals could survive on saline without any glucocorticoid replacement following the adrenalectomy^45^. We then performed blood collection from the rat heart and tissue sampling at the following designated periods. Sera were isolated by centrifugation at 1,500 × *g* for 30 min at 4 °C and stored at −80 °C until use.

### HE staining

Adrenal tissues were immersion-fixed overnight in 4% formaldehyde in phosphate-buffered saline (PBS), and frozen tissues, embedded in OCT compound, were cut into 10 µm-thick sections. Each 10 µm-thick frozen section was hydrated and stained with HE according to the general method.

### Immunohistochemistry

The procedure of fixing and sectioning were the same as the procedure in the section above. Heat-induced epitope retrieval was conducted with 10 mM Tris-HCl (pH 6.0) for 5 min for PCNA and DAB2 staining. Inactivation of endogenous peroxidases with 0.1% phenylhydrazine in PBS was performed for 30 min. Sections were then incubated with mouse anti-PCNA antibody (610664, BD biosciences, CA, USA; dilution at 1:1000) with PBS containing 0.3% triton X-100 or mouse anti-Disabled-2/p96 antibody (610464; BD biosciences, CA, USA; dilution at 1:1000) with PBS containing 0.3% triton X-100 overnight at room temperature. After washing with PBS containing 0.3% triton X-100, sections were incubated with ImmPRESS REAGENT anti-mouse IgG (MP-7422; VECTOR LABORATORIES, INC., CA, USA) for 30 min, followed by staining with diaminobenzidine. Sections were counterstained with haematoxylin.

For CYP11B2, heat-induced epitope retrieval step was omitted. After inactivation of endogenous peroxidases, sections were blocked with 10% goat serum and 0.5% SDS in 0.1 M Tris pH 7.4 for 1 h. Then, sections were incubated with rabbit anti-CYP11B2 antibody^24^ (dilution 1:6000 with 0.1 M Tris, 10% goat serum and 0.2% Tween-20) supplied by C. Gomez-Sanchez (University of Mississippi Medical Center) overnight at room temperature. After washing with PBS containing 0.2% Tween-20, sections were incubated with goat anti-rabbit IgG-Alexa flour 488 for 1 h (A27034; Thermo Fisher Scientific, MA, USA; dilution 1:1000 with 0.1 M Tris, 10% goat serum and 0.2% Tween-20).

Control experiments with omission of the antibodies were conducted to extract the reference spectral profile.

### Enzyme immunoassay for serum corticosterone and ACTH levels

We measured the rat serum corticosterone and ACTH levels in duplicate using a Corticosterone EIA Kit (YK240; Yanaihara Institute Inc., Shizuoka, Japan) and an ACTH ELISA kit (M046006; MD bioproducts, Zurich, Switzerland) following the manufacturer’s protocol, respectively. The range of detection for the corticosterone concentration was from 0.21 to 50 ng/mL and for ACTH concentration was from 1.0 to 640 pg/ml, respectively. The intra-assay coefficient of variation and inter-assay coefficient of variation were 2.5–4.7% and 7.7–9.8% in Corticosterone EIA Kit and 2.24–6.71% and 6.9–7.1% in ACTH ELISA kit, respectively.

### RNAscope and Basescope (*in situ* hybridisation)

Adrenal tissues were treated according to the Sample Preparation Technical Note for Fixed Frozen Tissue (ACD, Hayward CA, USA). In brief, fixed frozen tissues embedded in OCT compound were cut into 10 µm-thick sections and mounted on Superfrost Plus slides (Fisher Scientific, Waltham MA, USA) for RNA *in situ* hybridisation. These sections were air-dried at −20 °C and stored at −80 °C until use.

We performed RNAscope analysis with rat probes (Supplementary Table 1) to reveal the mRNA expression and localisation in adrenocortical autografts using the BaseScope Reagent Kit, RNAscope 2.5HD Reagent Kit (Single-plex, RED), or RNAscope 2-plex Reagent Kit (ACD) with the HybEZ Hybridisation system according to the kit manual. Each mRNA was detected with FastRed or FastGreen. Counterstaining was conducted with 33% Mayer’s haematoxylin or 50% Gill’s haematoxylin.

### Direct count of mRNA copies

Adrenal glands of sham-operated rats and adrenocortical autografts were quickly removed. The removed adrenal tissues were isolated from the surrounding fat and muscle tissues as much as possible, and total RNA was extracted using Sepasol-RNA I Super G reagent (Nacalai Tesque, Inc., Kyoto, Japan). *Cyp11b1* and *Cyp11b2* mRNAs in each sample were hybridised with the target probes (Supplementary Table 2) and reporter tags (Elements XT TagSet system, NanoString Technologies, Inc. N Seattle, WA). After trapping, the RNA copy number was counted in a digital analyser with the nCounter system (NanoString Technologies, Inc. N Seattle, WA) according to the provided manual. We analysed the data using nSolver analysis software (NanoString Technologies, Inc. N Seattle, WA). Each mRNA count was normalised to *Hprt1* as the internal control gene. Background subtraction was performed using the raw counts of RNase-free water.

### Microscope imaging

Optical images were collected using ECLIPSE E1000M (Nikon, Tokyo, Japan), ECLIPSE Ci (Nikon, Tokyo, Japan), or LSM700 (ZEISS, Oberkochen, Germany) microscopes. Autofluorescence frequency interferes with the identification of specific signals in fluorescence imaging and varies in each organ^46^. Some autofluorescence are derived from endogenous molecules related to metabolism, such as nicotinamide adenine dinucleotide phosphate, and lipofuscin^46–48^. Therefore, autofluorescence has been reported to be useful for monitoring metabolic changes^48^. The adrenal gland is one of the organs known to produce autofluorescence^47^. We found autofluorescence in the adrenal gland and adrenocortical autograft tissues (Supplementary Fig. 3). Recently, “unmixing” technique by spectral imaging was reported to separate desired signals from autofluorescence^49^. The autofluorescence spectral profile in adrenocortical cells could be clearly discriminated from the fluorescence with FastRed chromogen by RNAscope and from the fluorescence with Alexa 488 green fluorophore profile using lambda mode and spectral unmixing algorithms equipped with Zeiss LSM700 microscope. The autofluorescence spectral profile, as a reference, was extracted from the control experiments described above (Supplementary Fig. 4). Positive signal spectral profile of the fluorescence with FastRed chromogen in RNAscope was extracted from ovary tissues (Supplementary Fig. 4). For all the fluorescence images, we subtracted the autofluorescence from the original figures by linear unmixing.

### Statistical analysis

Normality was analysed by the Shapiro-Wilk normality test for all groups.

The distributions of the *Cyp11b1* and *Cyp11b2* expression levels via nCounter technology were compared between the sham and autograft groups at each time point using the Mann-Whitney *U*-test because the data from one group were not normally distributed (P < 0.05). P < 0.05 was considered to indicate a statistically significant difference in each comparison of gene expression. The distributions of corticosterone and ACTH concentrations between the sham and autograft groups were compared using ANOVA and the Mann-Whitney U test using SPSS for Windows. Significance levels were set at p < 0.0125 for corticosterone and ACTH concentrations after Bonferroni’s correction for multiple comparisons. The correlations between corticosterone and ACTH concentrations at each time were examined using the Spearman correlation coefficient test. A p value less than 0.05 was considered as statistically significant.

## Electronic supplementary material

Supplementary information

## References

[1] Ye P, Nakamura Y, Lalli E, Rainey WE (2009). Differential effects of high and low steroidogenic factor-1 expression on CYP11B2 expression and aldosterone production in adrenocortical cells. Endocrinology..

[2] Pihlajoki M, Dorner J, Cochran RS, Heikinheimo M, Wilson DB (2015). Adrenocortical zonation, renewal, and remodeling. Front Endocrinol (Lausanne)..

[3] Guasti L (2013). Dlk1 up-regulates Gli1 expression in male rat adrenal capsule cells through the activation of beta1 integrin and ERK1/2. Endocrinology..

[4] Mitani F (2014). Functional zonation of the rat adrenal cortex: the development and maintenance. Proc Jpn Acad Ser B Phys Biol Sci..

[5] Lenders JW (2014). Pheochromocytoma and paraganglioma: an endocrine society clinical practice guideline. J Clin Endocrinol Metab..

[6] Hahner S (2015). High incidence of adrenal crisis in educated patients with chronic adrenal insufficiency: a prospective study. J Clin Endocrinol Metab..

[7] Castinetti F (2016). MANAGEMENT OF ENDOCRINE DISEASE: Outcome of adrenal sparing surgery in heritable pheochromocytoma. Eur J Endocrinol..

[8] Inabnet WB, Caragliano P, Pertsemlidis D (2000). Pheochromocytoma: inherited associations, bilaterality, and cortex preservation. Surgery..

[9] Ingle DJ (1938). Autotransplantation and regeneration of the adrenal gland. Endocrinology..

[10] Elliott TR, Tuckett I (1906). Cortex and medulla in the suprarenal glands. J Physiol..

[11] Hurst AF, Tanner WE, Osman AA (1922). Addison’s Disease, with Severe Anaemia, treated by Suprarenal Grafting. Proc R Soc Med..

[12] Erdogan G (1994). Adrenal autotransplantation after total adrenalectomy: delayed determined function. Endocr J..

[13] Okamoto T (1996). Bilateral adrenalectomy with autotransplantation of adrenocortical tissue or unilateral adrenalectomy: treatment options for pheochromocytomas in multiple endocrine neoplasia type 2A. Endocr J..

[14] Belloni AS (1990). Investigations on the morphology and function of adrenocortical tissue regenerated from gland capsular fragments autotransplanted in the musculus gracilis of the rat. Endocrinology..

[15] Teebken OE, Scheumann GF (2000). Differentiated corticosteroid production and regeneration after selective transplantation of cultured and noncultured adrenocortical cells in the adrenalectomized rat. Transplantation..

[16] Freedman BD (2013). Adrenocortical zonation results from lineage conversion of differentiated zona glomerulosa cells. Dev Cell..

[17] Kataoka Y, Ikehara Y, Hattori T (1996). Cell proliferation and renewal of mouse adrenal cortex. J Anat..

[18] King P, Paul A, Laufer E (2009). Shh signaling regulates adrenocortical development and identifies progenitors of steroidogenic lineages. Proc Natl Acad Sci USA.

[19] Huang CC, Miyagawa S, Matsumaru D, Parker KL, Yao HH (2010). Progenitor cell expansion and organ size of mouse adrenal is regulated by sonic hedgehog. Endocrinology..

[20] Laufer E, Kesper D, Vortkamp A, King P (2012). Sonic hedgehog signaling during adrenal development. Mol Cell Endocrinol..

[21] Finco I, LaPensee CR, Krill KT, Hammer GD (2015). Hedgehog signaling and steroidogenesis. Annu Rev Physiol..

[22] Wood MA (2013). Fetal adrenal capsular cells serve as progenitor cells for steroidogenic and stromal adrenocortical cell lineages in M. musculus. Development..

[23] Wang F (2012). RNAscope: a novel *in situ* RNA analysis platform for formalin-fixed, paraffin-embedded tissues. J Mol Diagn..

[24] Wotus C, Levay-Young BK, Rogers LM, Gomez-Sanchez CE, Engeland WC (1998). Development of Adrenal Zonation in Fetal Rats Defined by Expression of Aldosterone Synthase and 11beta-Hydroxylase. Endocrinology..

[25] Romero DG (2007). Disabled-2 is expressed in adrenal zona glomerulosa and is involved in aldosterone secretion. Endocrinology..

[26] Tanaka R (2011). Accurate determination of S-phase fraction in proliferative cells by dual fluorescence and peroxidase immunohistochemistry with 5-bromo-2′-deoxyuridine (BrdU) and Ki67 antibodies. J Histochem Cytochem..

[27] Guasti L, Paul A, Laufer E, King P (2011). Localization of Sonic hedgehog secreting and receiving cells in the developing and adult rat adrenal cortex. Molecular and Cellular Endocrinology..

[28] Bandiera R (2013). WT1 maintains adrenal-gonadal primordium identity and marks a population of AGP-like progenitors within the adrenal gland. Dev Cell..

[29] Till H, Metzger R, Mempel T, Boehm R, Joppich I (2000). Proliferation, zonal maturation, and steroid production of fetal adrenal transplants in adrenalectomized rats. Pediatr Surg Int..

[30] Engeland WC, Gomez-Sanchez CE, Fitzgerald DA, Rogers LM, Holzwarth MA (1996). Phenotypic changes and proliferation of adrenocortical cells during adrenal regeneration in rats. Endocr Res..

[31] Sarria R, Losada J, Bueno-Lopez JL (1995). Immunohistochemical analysis of adrenal proliferation and corticosterone expression in experimental adrenal regeneration. Histol Histopathol..

[32] Belloni AS (1982). Ultrastructural observations on the regeneration of adrenocortical autotransplants in the rat spleen. J Anat..

[33] Finkielstein CV, Capelluto DG (2016). Disabled-2: A modular scaffold protein with multifaceted functions in signaling. Bioessays..

[34] Tao W, Moore R, Smith ER, Xu XX (2014). Hormonal induction and roles of Disabled-2 in lactation and involution. Plos One..

[35] Boulkroun S (2010). Adrenal cortex remodeling and functional zona glomerulosa hyperplasia in primary aldosteronism. Hypertension..

[36] Varjosalo M, Taipale J (2008). Hedgehog: functions and mechanisms. Genes Dev..

[37] Franco HL, Yao HH (2012). Sex and hedgehog: roles of genes in the hedgehog signaling pathway in mammalian sexual differentiation. Chromosome Res..

[38] Bandiera R, Sacco S, Vidal VP, Chaboissier MC, Schedl A (2015). Steroidogenic organ development and homeostasis: A WT1-centric view. Mol Cell Endocrinol..

[39] Scholz H, Kirschner KM (2011). Oxygen-Dependent Gene Expression in Development and Cancer: Lessons Learned from the Wilms’ Tumor Gene, WT1. Front Mol Neurosci..

[40] Wagner KD (2014). The Wilms’ tumour suppressor Wt1 is a major regulator of tumour angiogenesis and progression. Nat Commun..

[41] McCarty G, Awad O, Loeb DM (2011). WT1 protein directly regulates expression of vascular endothelial growth factor and is a mediator of tumor response to hypoxia. J Biol Chem..

[42] Kirschner KM, Sciesielski LK, Krueger K, Scholz H (2017). Wilms tumor protein-dependent transcription of VEGF receptor 2 and hypoxia regulate expression of the testis-promoting gene Sox9 in murine embryonic gonads. J Biol Chem..

[43] Taniguchi A (2004). Expression of vascular endothelial growth factor and its receptors Flk-1 and Flt-1 during the regeneration of autotransplanted adrenal cortex in the adrenalectomized rat. J Urol..

[44] Takizawa, N. *et al*. Hypothalamohypophysial system in rats with autotransplantation of the adrenal cortex. *Mol Med Rep* (2017). 10.3892/mmr.2017.6375 28339047

[45] Srougi M, Gittes RF, Underwood RH (1980). Influence of exogenous glucocorticoids and ACTH on experimental adrenal autografts. Invest Urol..

[46] Jun YW, Kim HR, Reo YJ, Dai M, Ahn KH (2017). Addressing the autofluorescence issue in deep tissue imaging by two-photon microscopy: the significance of far-red emitting dyes. Chem Sci..

[47] Schonenbrucher H (2008). Fluorescence-based method, exploiting lipofuscin, for real-time detection of central nervous system tissues on bovine carcasses. J Agric Food Chem..

[48] Santin G (2013). Autofluorescence properties of murine embryonic stem cells during spontaneous differentiation phases. Lasers Surg Med..

[49] Levenson R, Beechem J, McNamara G (2012). Spectral imaging in preclinical research and clinical pathology. Anal Cell Pathol (Amst)..

