# Supplementary material for: Involvement of DHH and GLI1 in adrenocortical autograft regeneration in rats
Source: Sci Rep. 2018 Sep 28;8:14542. doi: 10.1038/s41598-018-32870-9 (PMC6162278; doi:10.1038/s41598-018-32870-9)

Supplementary Information

Involvement of DHH and GLI1 in adrenocortical autograft regeneration in rats

Nae Takizawa, Susumu Tanaka, Souichi Oe, Taro Koike, Takashi Yoshida,

Yukie Hirahara, Tadashi Matsuda and Hisao Yamada.

Supplementary Table 1. Probes for Basescope and RNAscope

Gene symbol	Accession No.	Probe Cat No.	Number of ‘ZZ’ probe binding sites	Target Region
Cyp11b1	NM_012537.3	710341	4	435 — 658
Shh	NM_017221.1	501541-C2	20	68 — 1654
Dhh	NM_053367.1	494681	20	1254 — 2476
Ihh	NM_053384.1	494671	20	1094 — 2067
Gli1	NM_001191910.1	435581-C2	20	1044 — 2068
Wt1	NM_031534.2	429891	20	418 — 1494

Supplementary Table 2. Target specific probes for nCounter

Gene symbol	Accession No	Position	Target Sequence
Cyp11b1	NM_012537.3	1077 - 1176	AGACCTGCCCTTGCTGCGGGCTG CCCTTAAAGAGACCTTGAGGCTCT ACCCTGTTGGTAGCTTTGTAGAGA GAATCGTACACTCAGACCTGGTGC TTCAG
Cyp11b2	NM_012538.2	856 - 955	CAGACCTACAGTGGCATTGTGGC AGCACTAATAACTCAGGGAGCTTT ACCTCTGGACGCCATCAAAGCCA ACTCTATGGAGCTCACTGCTGGGA GCGTTG
Hprt1	NM_012583.2	21 - 120	AGCTTCCTCCTCAGACCGCTTTTC CCGCGAGCCGACCGGTTCTGTCAT GTCGACCCTCAGTCCCAGCGTCG TGATTAGTGATGATGAACCAGGTT ATGAC

Supplementary Figure 1.

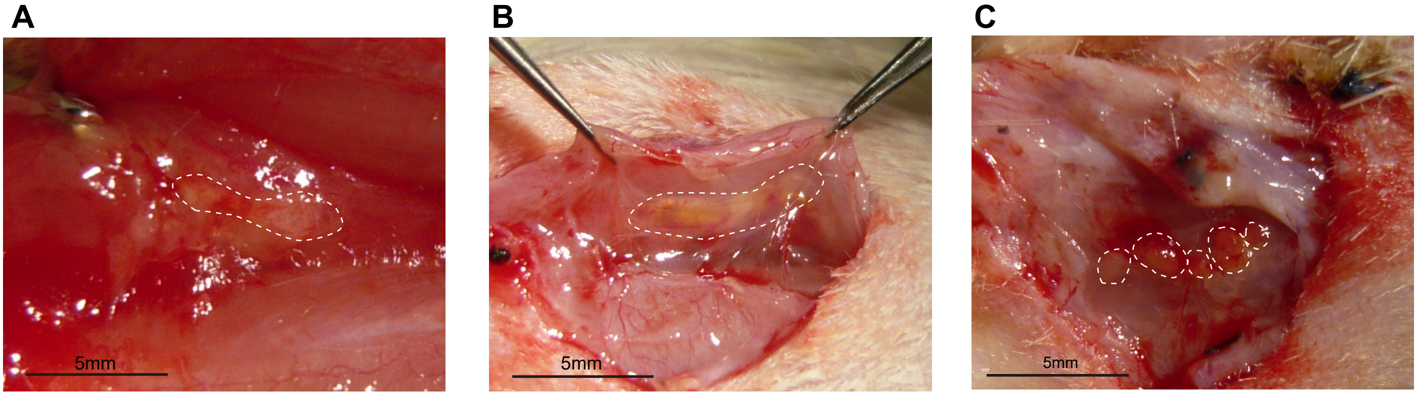

Macroscopic findings of adrenocortical autografts.

(A), (B), and (C) show the gross appearance of adrenocortical autografts at POD7, 14, and 21, respectively. The white broken line circle shows the adrenal autograft in the right biceps femoris. Scale bars = 5mm.

Supplementary Figure 2.

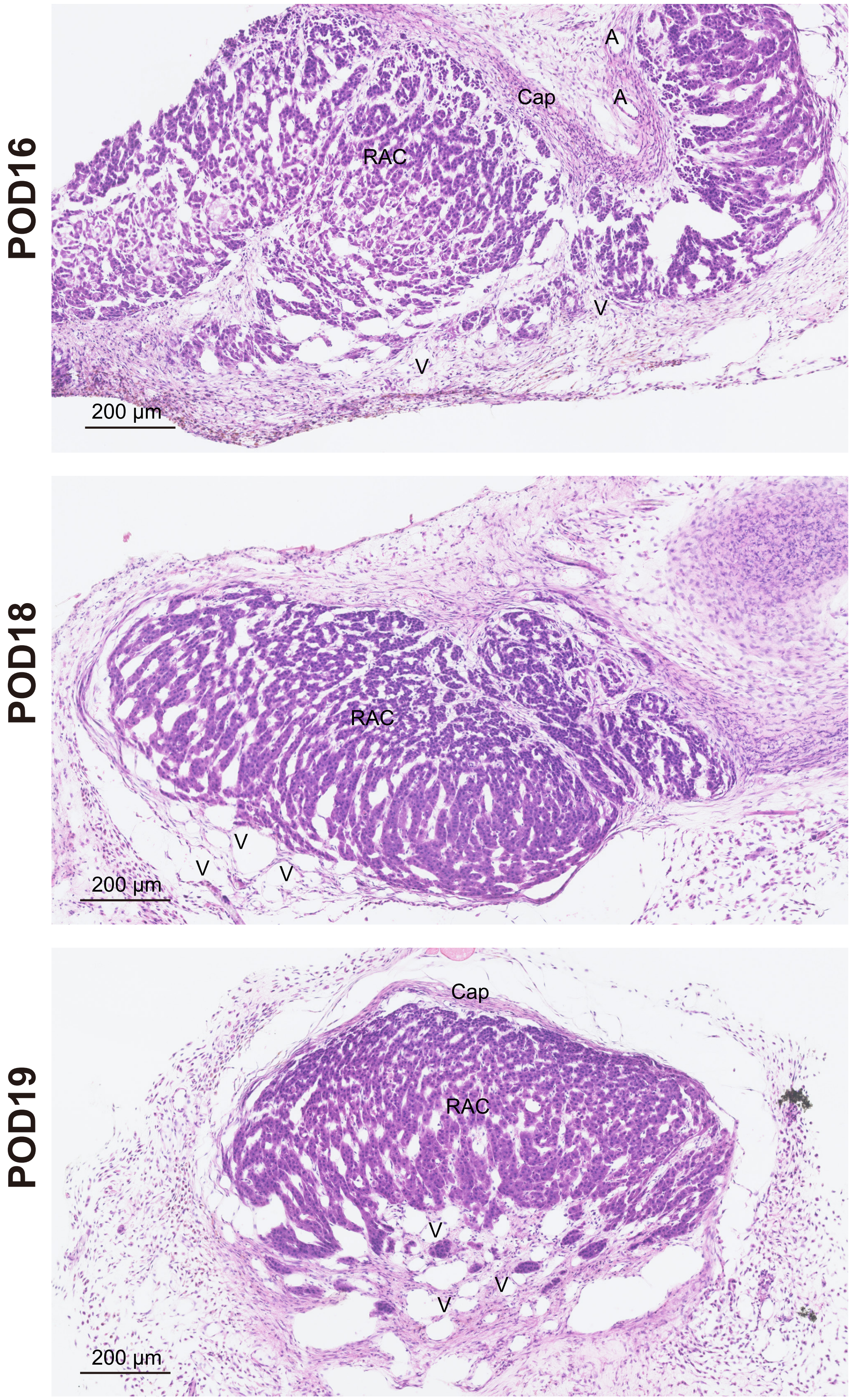

Sections of adrenocortical autografts from post-operative days 16-19 stained with haematoxilin and eosin.

C: capsule; RAC: renewal adrenocortical cell; A: artery; V: vein. Scale bars = 200μm.

Supplementary Figure 3.

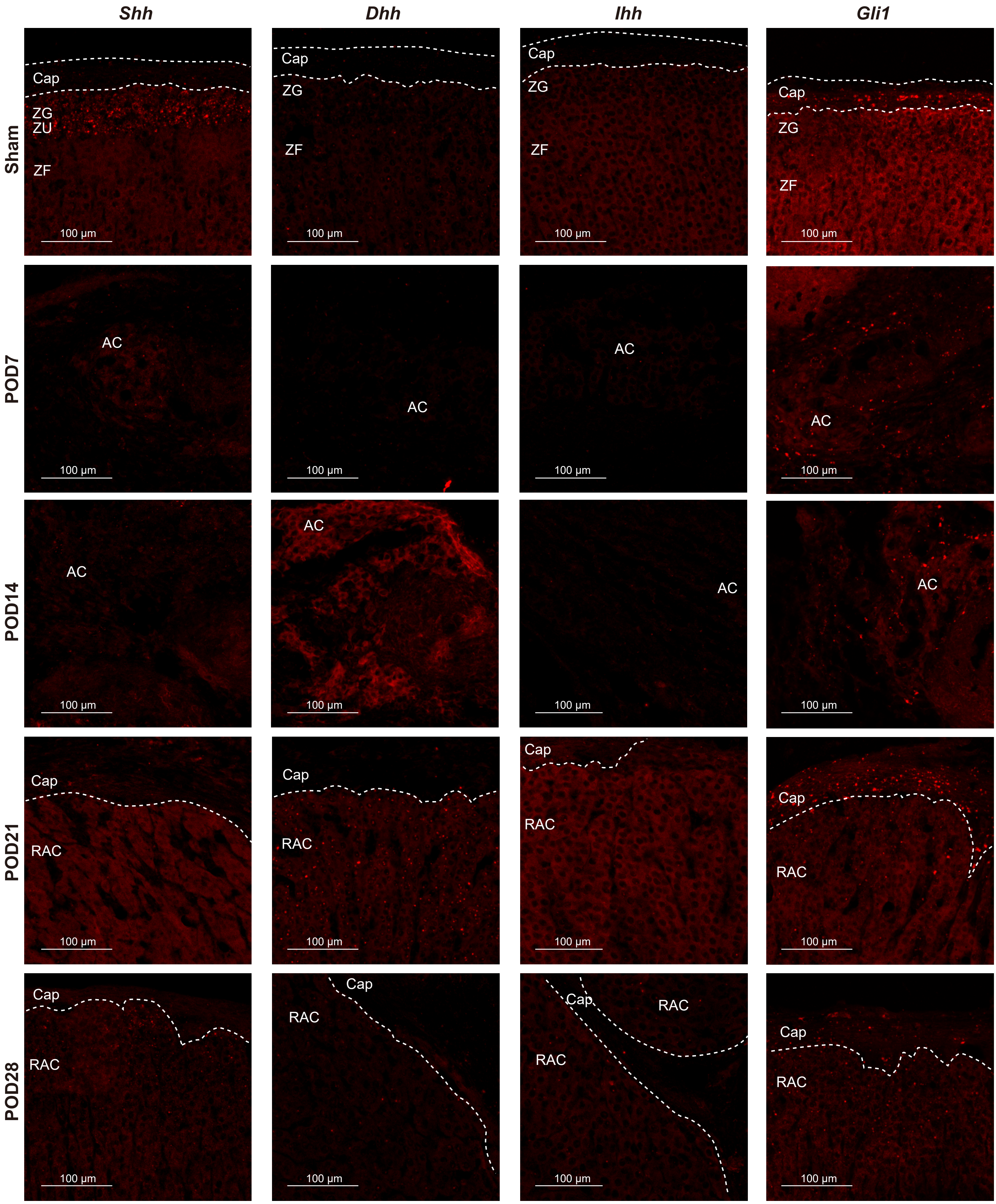

Original (pre-unmixing) images of Hh signalling pathway in sham-operated adrenal gland and adrenocortical autografts. Cap: capsule; ZG: zona glomerulosa; ZU: undifferentiated zone; ZF: zona fasciculata; AC: adrenocortical cells; RAC: renewal adrenocortical cells.

The white broken line shows the boundary between the adrenal capsule and adrenocortical cells.

Supplementary Figure 4.

Reference spectral profiles for unmixing of RNAscope signal from autofluorescence in adrenal gland or adrenocortical autogurafts.

(A) – (E) represent control slides in each period. (F) represents the positive control slide, which is the ovary tissue with *Dhh*-targeted probe.

A **Adrenal gland**

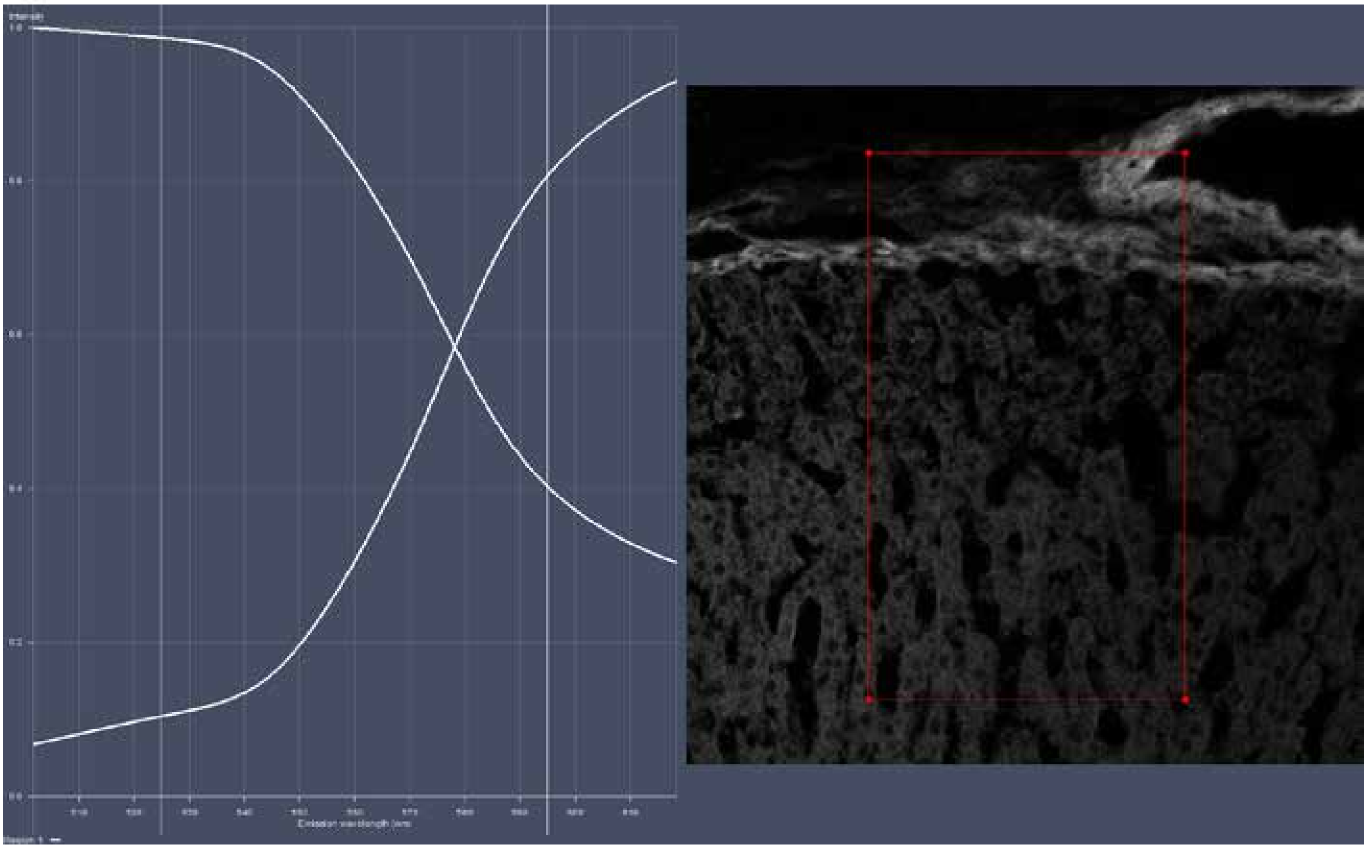

B **The adrenocortical autograft at POD7**

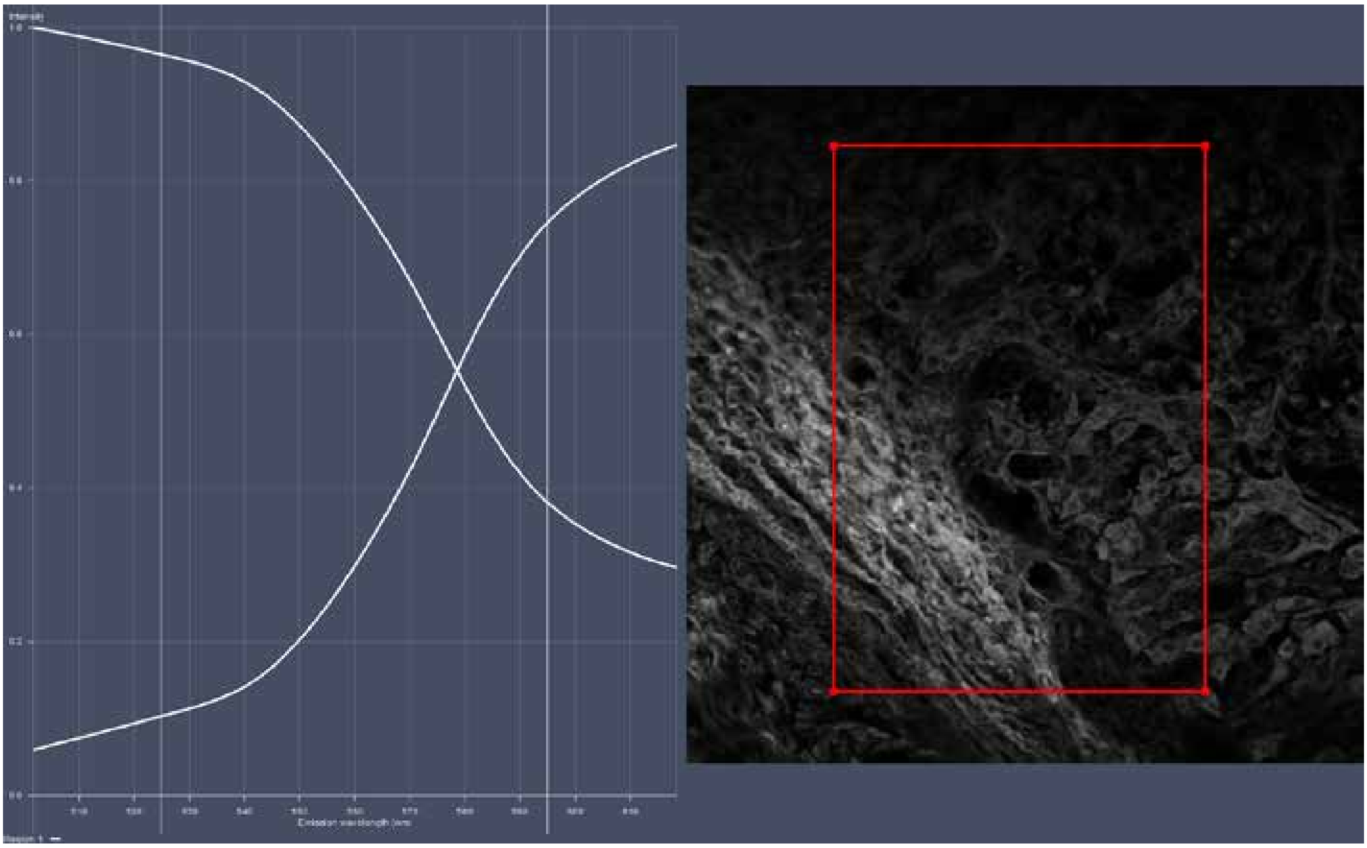

C The adrenocortical autograft at POD14

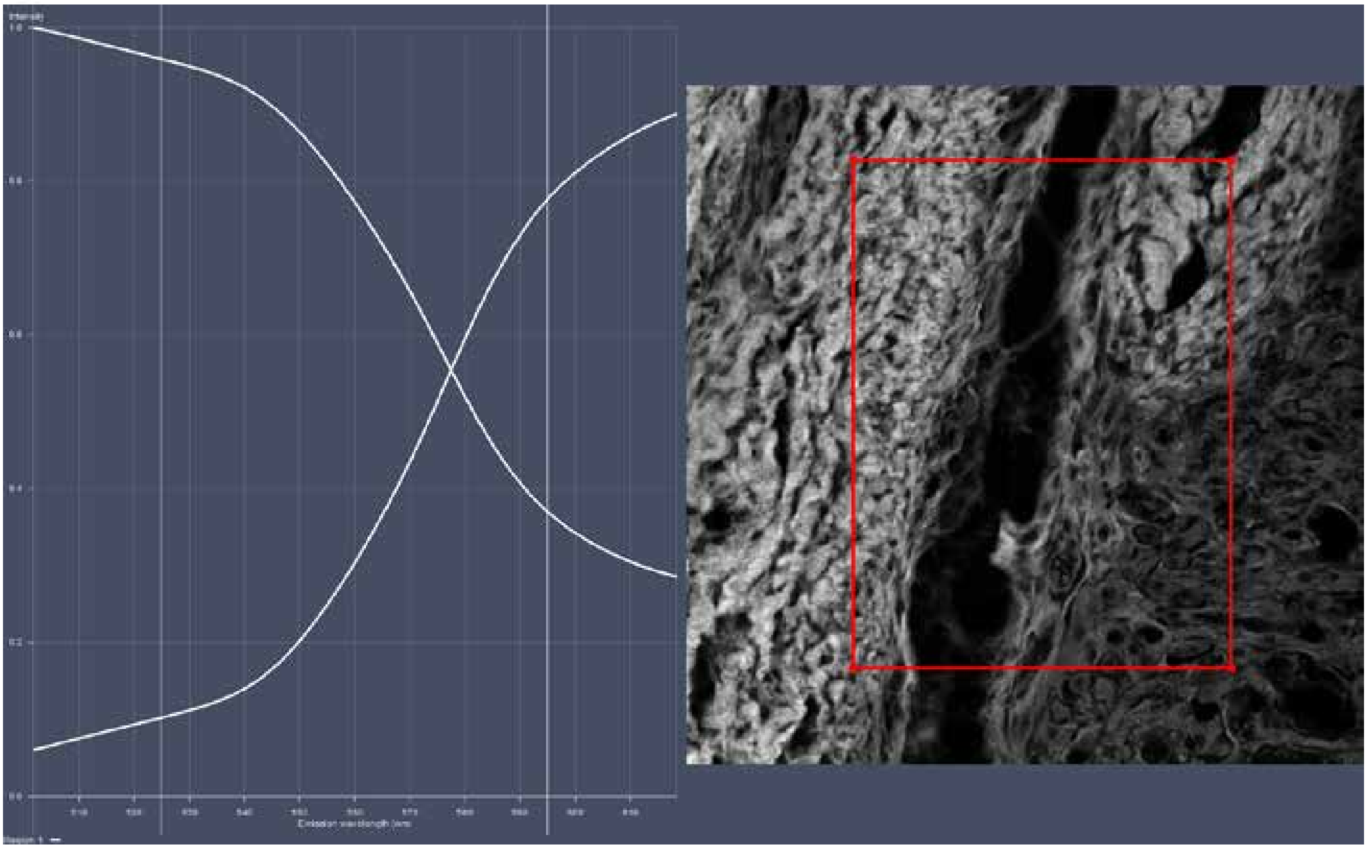

D The adrenocortical auutograft at POD21

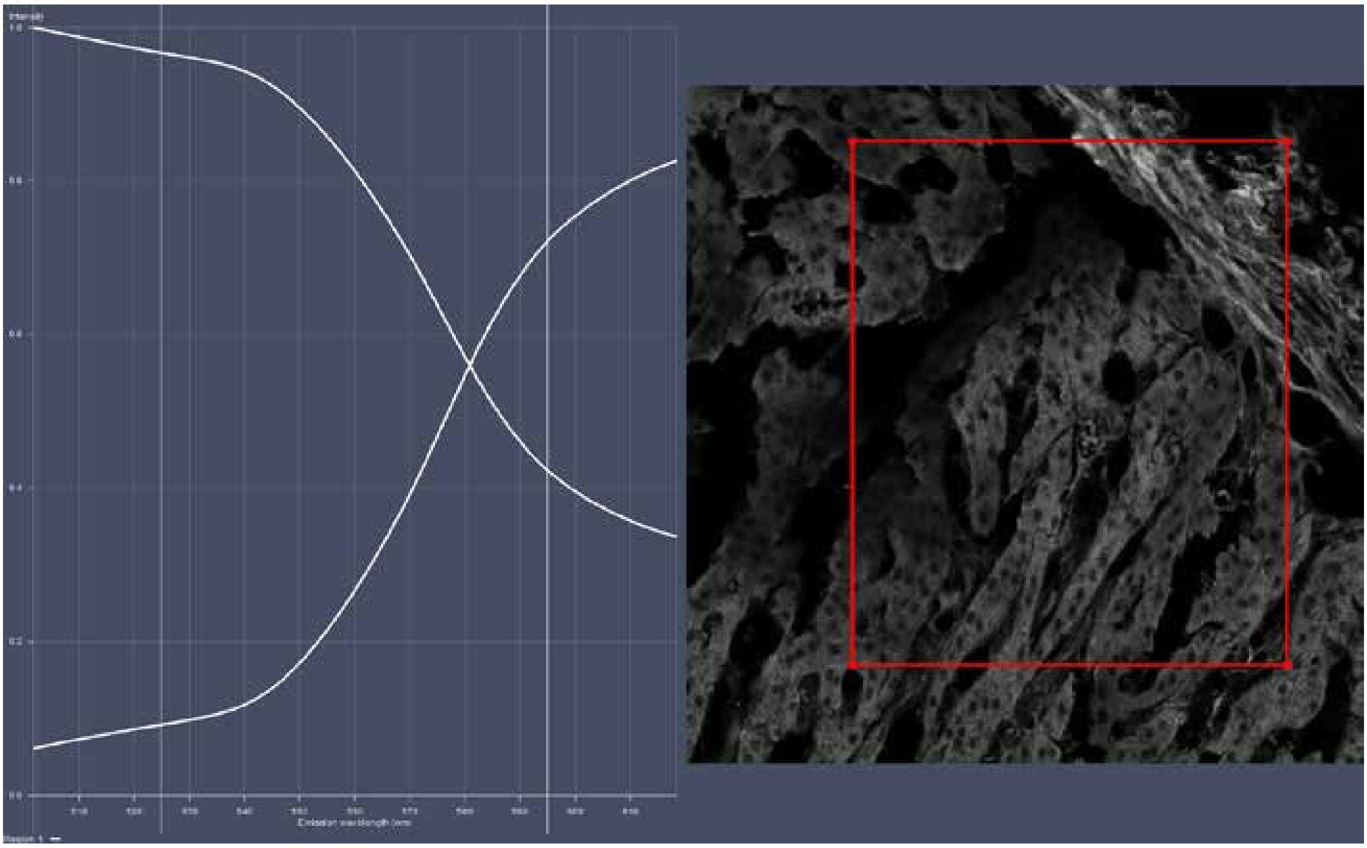

E **The adrenocortical autograft at POD28**

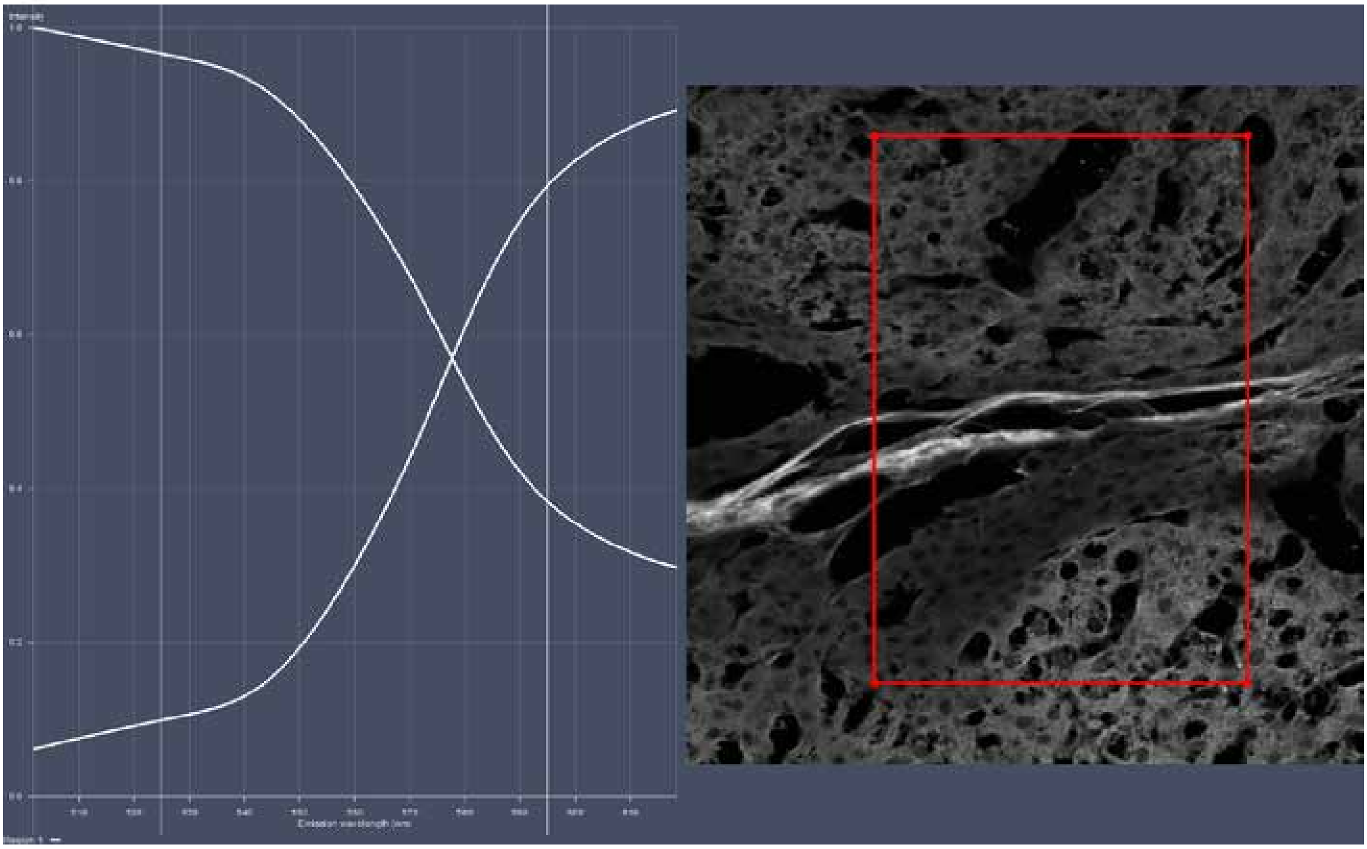

F **Ovary tissue**

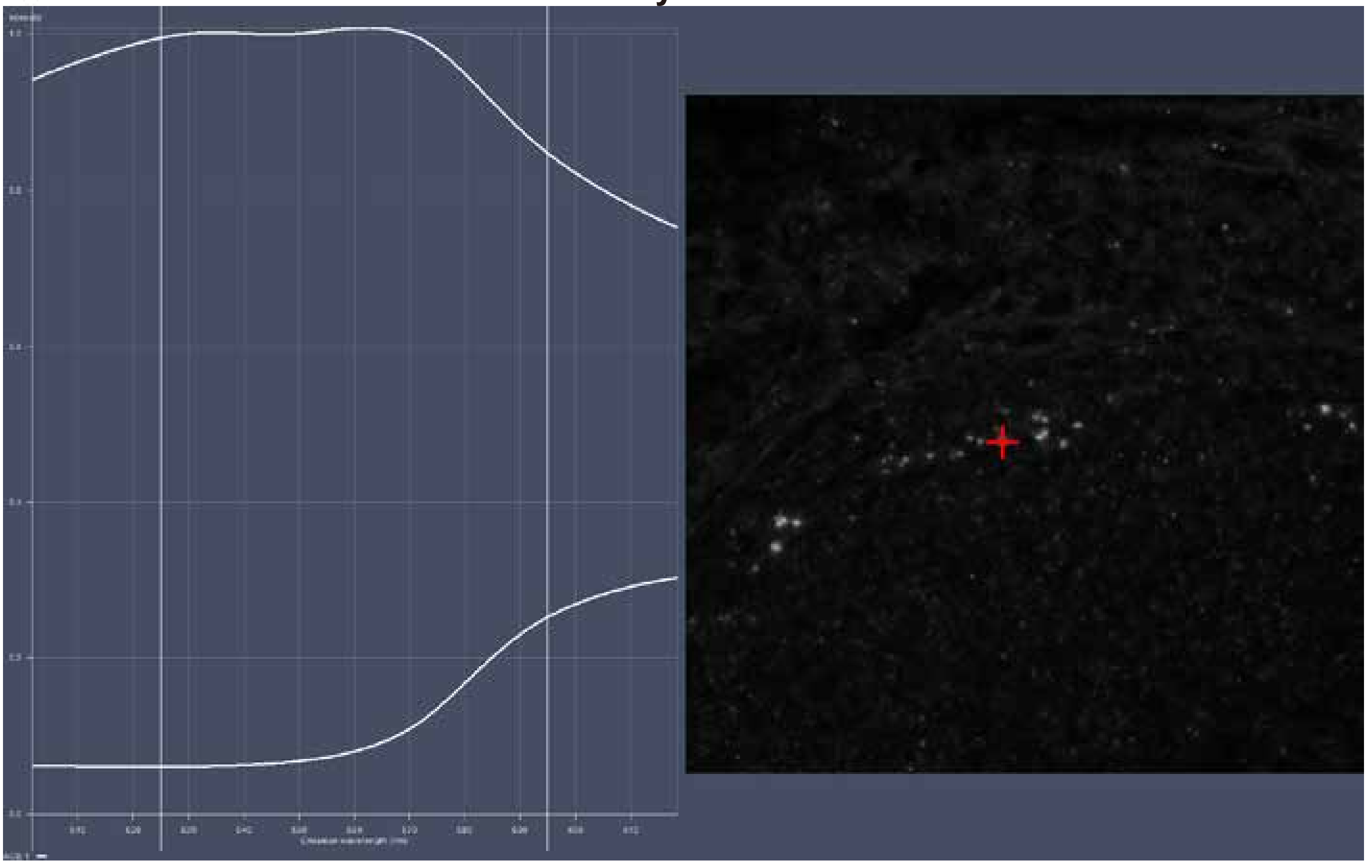

Supplement: Supplementary file 1 — Supplementary information [file 41598_2018_32870_MOESM1_ESM.pdf]
